# Effect of Mobile Health Interventions on Lifestyle and Anthropometric Characteristics of Uncontrolled Hypertensive Participants: Secondary Analyses of a Randomized Controlled Trial

**DOI:** 10.3390/healthcare11081069

**Published:** 2023-04-08

**Authors:** Caroline Nespolo David, Cirano Iochpe, Erno Harzheim, Guilhermo Prates Sesin, Marcelo Rodrigues Gonçalves, Leila Beltrami Moreira, Flavio Danni Fuchs, Sandra Costa Fuchs

**Affiliations:** 1 Postgraduate Studies Program in Epidemiology, School of Medicine, Universidade Federal do Rio Grande do Sul, Porto Alegre 90010-150, Brazil; 2 Informatics Institute, Universidade Federal do Rio Grande do Sul, Porto Alegre 90010-150, Brazil; 3 Postgraduate Studies Program in Cardiology, Hospital de Clínicas de Porto Alegre, School of Medicine, Universidade Federal do Rio Grande do Sul, Porto Alegre 90010-150, Brazil

**Keywords:** hypertension, lifestyle, physical activity, diet, body fat, digital health, mHealth

## Abstract

Our objective was to evaluate the effect of a mobile health (mHealth) intervention on lifestyle adherence and anthropometric characteristics among individuals with uncontrolled hypertension. We performed a randomized controlled trial (ClinicalTrials.gov NCT03005470) where all participants received lifestyle counseling at baseline and were randomly allocated to receive (1) an automatic oscillometric device to measure and register blood pressure (BP) via a mobile application, (2) personalized text messages to stimulate lifestyle changes, (3) both mHealth interventions, or (4) usual clinical treatment (UCT) without technology (control). The outcomes were achieved for at least four of five lifestyle goals (weight loss, not smoking, physical activity, moderate or stopping alcohol consumption, and improving diet quality) and improved anthropometric characteristics at six months. mHealth groups were pooled for the analysis. Among 231 randomized participants (187 in the mHealth group and 45 in the control group), the mean age was 55.4 ± 9.5 years, and 51.9% were men. At six months, achieving at least four of five lifestyle goals was 2.51 times more likely (95% CI: 1.26; 5.00, *p* = 0.009) to be achieved among participants receiving mHealth interventions. The between-group difference reached clinically relevant, but marginally significant, reduction in body fat (−4.05 kg 95% CI: −8.14; 0.03, *p* = 0.052), segmental trunk fat (−1.69 kg 95% CI: −3.50; 0.12, *p* = 0.067), and WC (−4.36 cm 95% CI: −8.81; 0.082, *p* = 0.054), favoring the intervention group. In conclusion, a six-month lifestyle intervention supported by application-based BP monitoring and text messages significantly improves adherence to lifestyle goals and is likely to reduce some anthropometric characteristics in comparison with the control without technology support.

## 1. Introduction

High blood pressure (BP) is a primary cause of cardiovascular diseases [1]. The prevalence of individuals with BP of 140/90 mmHg or higher has doubled since 1990, reaching 626 million women and 652 million men in 2019, and less than half on treatment had controlled hypertension [2]. However, a healthy lifestyle and BP-lowering medication can avert BP elevation [3], leading to reduced mortality rates, years lived with disability, and years of life lost [4].

A healthy lifestyle can aid in controlling hypertension. This includes maintaining a normal weight, following a healthy low-sodium diet, increasing physical activity, quitting smoking, and limiting alcohol consumption [3,5]. Additionally, a healthy lifestyle can prevent excess body fat accumulation and its associated risk of uncontrolled hypertension [6,7,8]. Despite a diagnosis of hypertension, few patients tend to modify their lifestyle or sustain adherence to lifestyle recommendations [9,10,11].

Health technology has opened up new opportunities for enhancing care for chronic conditions, such as hypertension [12,13]. This is particularly relevant after the COVID-19 pandemic. Given the ever-increasing number of smartphone users worldwide [14], mHealth interventions have the potential to not only control BP, but also foster significant lifestyle modifications [15]. Despite numerous randomized controlled trials evaluating the effectiveness of mHealth for promoting smoking cessation, physical activity, dietary improvement, alcohol consumption reduction, and weight loss, the findings have been inconclusive. The results are influenced by various factors, including population characteristics, type of technology, lifestyle intervention, follow-up duration, health professional involvement, and comparator utilized [12,16,17]. Therefore, further studies are required to assess the effectiveness of diverse mHealth strategies in enhancing risk factor management and overall lifestyle. 

We evaluated two mHealth interventions and their effectiveness compared to the usual standard of care in outpatient clinics. Specifically, we examined their impact on risk factors for hypertension. If the results were significant, it may be feasible to apply these interventions to outpatients of public health. This analysis aims to determine whether a six-month lifestyle intervention, supported by mHealth, can improve the lifestyle and reduce anthropometric indices of individuals with uncontrolled hypertension who are taking BP-lowering medications.

## 2. Materials and Methods

### 2.1. Design

This factorial randomized controlled trial was designed to evaluate Technologies for Innovative Monitoring (the TIM Study) to reduce BP (primary endpoint) and change lifestyle (secondary endpoint). Participants were randomly assigned to one of four groups: (1) telemonitoring home BP (TELEM), (2) text messages for lifestyle (TELEMEV), (3) telemonitoring home BP, and text messages for lifestyle (TELEM-TELEMEV), or (4) control (usual clinical treatment [UCT]). The purpose of TIM Study was to explore potential interventions for promoting lifestyle changes, body fat and/or reducing blood pressure. While the specific method used may not be critical for public health purposes, the intervention must be effective. To that end, we combined the three intervention groups into a pooled mHealth intervention for analysis, while also presenting effect sizes and confidence intervals for each intervention arm separately. The TIM study is registered at www.clinicaltrials.gov with the ID number: NCT03005470 and details have been published [18].

### 2.2. Participants

Participants were recruited from primary care clinics or online advertising. The trial enrolled individuals aged 30 to 75 years, with hypertension diagnosis, taking one or two BP-lowering medications, and uncontrolled BP. Participants must have a smartphone and internet access. To determine eligibility for the study, we conducted face-to-face consultations with participants and evaluated their blood pressure using standard protocols. To be eligible, participants had to meet two criteria: first, their office blood pressure had to be uncontrolled, which was defined as a systolic blood pressure of 135 mmHg or higher or a diastolic blood pressure of 85 mmHg or higher. Second, their ambulatory blood pressure monitoring had to be uncontrolled, which was defined as a 24 h systolic blood pressure of 130 mmHg or higher or a diastolic blood pressure of 80 mmHg or higher.

We excluded participants with severe hypertension (systolic BP ≥ 180 mmHg or diastolic BP ≥ 110 mm Hg), a major cardiovascular event in the previous six months, other indications for the use of antihypertensive medication, diagnosis of secondary hypertension, pregnancy or lactation, or inability to tolerate the interventions. In three consecutive morning office visits, potentially eligible participants were evaluated to confirm BP eligibility criteria. The study was conducted in the Clinical Research Center of the Hospital de Clínicas de Porto Alegre. The institution’s institutional review board approved the study (GPPG number 16-0187/CAAE 31423214.0.0000.5327), and written informed consent was obtained from all participants according to the principles expressed in the Declaration of Helsinki.

### 2.3. Interventions and Control

All participants received an individual personalized lifestyle session at baseline. The lifestyle session was performed by a certified researcher with the support of an illustrated colorful booklet, where we presented specific recommendations for living a better lifestyle to control their BP. The instructions included maintaining a normal weight, following the dietary approach to stop hypertension (DASH-type diet) with low sodium intake, performing regular physical activity, stopping smoking or maintaining a habit of not smoking, and no drinking or moderate consumption of alcohol. The study arms are described below.

#### 2.3.1. TELEM Intervention

Participants received an automatic oscillometric device to measure BP five days per week and one day on the weekend. Participants were trained to use the monitor and instructed to perform four daily measurements (two in the morning and two in the evening). The monitor sent BP values to the data center through an application. Participants could also enter BP data manually in the application. After sending BP measurements, participants received a prompt on the mobile phone with feedback about their BP control based on the value entered.

#### 2.3.2. TELEMEV Intervention

Participants received personalized, standardized unidirectional text messages via an application developed for the study to stimulate lifestyle changes. Experts developed messages based on guidelines that emphasized the adoption of a DASH-type diet, reducing sodium intake, reducing alcohol consumption, increasing physical activity, managing weight loss, and taking medications to lower blood pressure regularly. These messages were inserted into software which sent messages automatically, four days a week, during random business hours, without another cell phone contact. Examples of text messages can be found in the Supplementary Materials (Table S1).

#### 2.3.3. TELEM-TELEMEV Intervention

Participants received both mHealth interventions: telemonitoring of BP plus text messages via a mobile application.

#### 2.3.4. UCT

The control group received the healthy lifestyle intervention using the information presented in the booklet in a guided session. Participants did not receive any technological tools or additional BP control.

We combined the three intervention groups into a pooled mHealth intervention for this analysis. Considering that the effectiveness of any intervention would allow for achieving the objective of the study, the lack of interaction between the intervention arms and the outcomes as well as the limited power to assess the individual arm’s effect on lifestyle support this approach.

From randomization, follow-ups were scheduled for 7, 30, 90, and 180 days. Participants were asked about adherence to lifestyle recommendations during all visits and were allowed to clarify doubts with the researchers.

### 2.4. Measurements

Demographic and socioeconomic characteristics were collected at baseline, including age, sex, reported skin color, education level, and work status. All measurements taken during the study were performed on calibrated equipment. At baseline and six-month follow-up, standardized anthropometric measurements of waist circumference (WC), at the midpoint between the lower costal margin and the iliac crest, body weight (kg), and height (cm), to calculate the body mass index (BMI, in kg/m^2^), were collected at the clinic by certified healthcare professionals. The bioelectrical impedance analysis InBody 230 (Biospace Co., Ltd., Des Moines, IA, USA) estimated total and segmental trunk body fat. Physical activity level was evaluated using the short-form International Physical Activity Questionnaire [19] for the seven days prior to the randomization, as well as dietary consumption using a validated Food Frequency Questionnaire [20]. Alcohol consumption and smoking status were evaluated using standardized questionnaires. This data collection was repeated in the follow-up.

### 2.5. Outcomes

The lifestyle change was operationalized by achieving four out of five goals in the follow-up without medication titration. The five lifestyle goals were as follows: lose at least 3 kg or maintain normal BMI; refrain from smoking; engage in regular physical activity for at least 150 min/week; consume no alcohol or drink moderately (≤100 g and ≤200 g of alcohol/week for women and men, respectively); achieve at least two of six dietary recommendations: reach or maintain at least 21 servings/week of fruits and vegetables, 14 servings/week of whole grains, seven servings/week of low-fat dairy; consume only one serving/week of sodium-rich foods, <1 serving/week of fast foods or fried foods, and <1 serving/week) of fried or fatty meats. The reduction of at least one anthropometric characteristic was considered a successful outcome (i.e., body fat mass [kg], percentage of body fat [%], segmental trunk fat [kg], percentage of segmental trunk fat [%], BMI [kg/m^2^], or WC [cm]).

### 2.6. Randomization

A computer-generated sequence was created using random allocation software [21] to assign participants to groups using permuted random block sizes of four and eight. An investigator not involved in the participants’ enrollment generated the randomization sequence before the trial began. The sequence list was kept in Research Electronic Data Capture software [22], preventing the research team from anticipating to which arm the next participant would be allocated. Data were released after the baseline data collection was completed. Due to the nature of the study, subjects could not be blinded to the intervention; however, the investigator who performed the data analysis was blinded.

### 2.7. Statistical Methods

TIM Study sample size was calculated to detect a reduction of systolic BP assessed by 24 h ABPM (primary endpoint), and the results were previously presented [23]. This analysis presented data from the secondary endpoint defined a priori. The recruitment of 231 subjects (with 186 in one of the mHealth interventions groups and 45 in control) had sufficient statistical power (85%) to detect a statistically significant difference in 25% achievement of the four of five lifestyle goals between groups with a significance level of 0.05 using a two-sided test. The effectiveness of the pooled mHealth intervention (TELEM + TELEMEV + TELEM-TELEMEV groups) was compared to the control group, which did not receive mHealth intervention (UCT group). Independent samples *t*-test for continuous and chi-square for categorical variables were used to describe the baseline characteristics. A generalized Poisson mixed model was used for binary outcomes of achieving lifestyle goals between mHealth and control groups, adjusting for baseline values, with relative risks and 95% confidence intervals (CIs). General Linear models (GLMs) were used to calculate weight and food groups consumption mean difference between-groups, adjusted for baseline value. Poisson and GLM analysis were performed using complete cases at 6 months. Generalized Estimating Equations (GEE) were used to analyze measurements taken over time on the same individuals. The response variable was the pooled mHealth intervention effect, which has a normal distribution. The identity link function was used. This assumption implies that the data points are not independent, and the GEE model takes this into account while analyzing the data. Additionally, the GEE model accounted for correlated data using an unstructured correlation matrix and adjusted for this correlation using robust covariance estimation. In the GEE, differences between the two groups at six months were evaluated using the adjustment of Bonferroni. These analyses were performed using the intention-to-treat approach in SPSS version 21.0; a two-sided *p* < 0.05 was considered statistically significant.

## 3. Results

**Figure 1:**
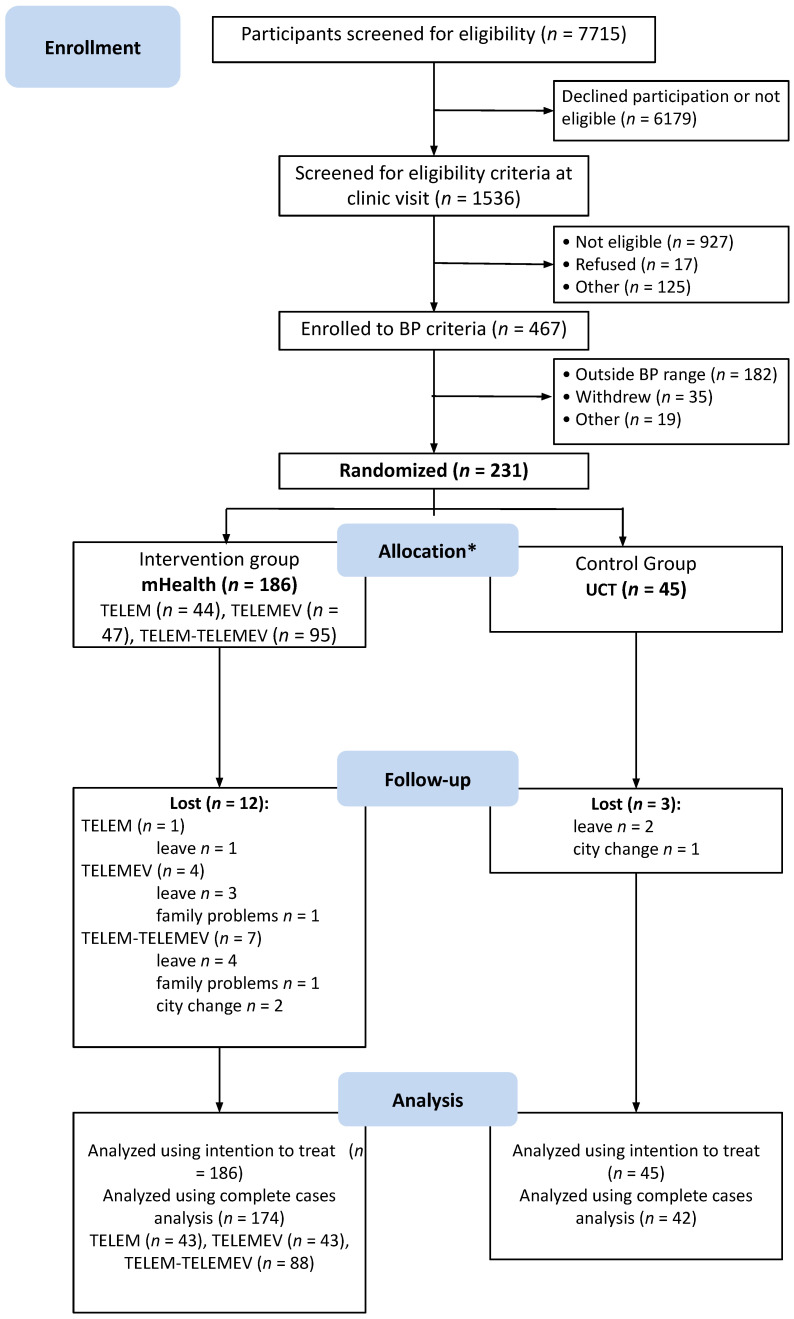
Study flowchart describing selection, randomization, and follow-up process. BP indicates blood pressure. * All participants received the allocated intervention.

From July 2016 to July 2018, 7750 potential participants were identified at the primary care facilities and the media announcements were screened; 1536 were checked to confirm eligibility at the research clinic. Of these, 467 had BP eligibility criteria assessed by ABPM, 231 met both office BP and ABPM criteria and were randomized. Participants were allocated to the pooled mHealth intervention or UCT control group at a ratio of 186 to 45 (Figure 1).

At the six-month follow-up, 174 participants in the intervention and 42 in the control group were evaluated for lifestyle characteristics. During follow-up, two participants in the pooled mHealth group discontinued the intervention (one from TELEM and one from TELEM-TELEMEV group) informing unavailability of time to perform the BP check as requested by the protocol. However, they followed up at the study visits. No adverse events were reported during the study follow-up. Table 1 shows that baseline characteristics were similar between groups. Participants were 55.4 ± 9.5 years (mean ± SD), 51.9% were men, and 64.9% were white, with mean education years of 11.1 ± 3.9, and 31% had not completed high school. The mean BMI was 30.5 ± 5.1 kg/m^2^, and the mean systolic and diastolic BP were 143.7 ± 11.4 mmHg and 89.6 ± 8.1 mm Hg, respectively.

### 3.1. Lifestyle Goals

Table 2 describes baseline and six-month lifestyle characteristics by intervention groups, as well as the relative risk (95% CI) at the end of the trial, adjusted for baseline values. At 6 months, participants in the pooled mHealth group were 1.2 (95% CI: 1.03; 1.42) times more likely to practice at least 150 min/week of physical activity at the end of the trial compared to the control group. In addition, the pooled mHealth group was 1.12 (95% CI: 1.00; 1.25) and 1.22 (95% CI: 1.04; 1.42) times more likely to moderate or no alcohol intake and improve diet quality at the end of the trial compared to the control group, respectively. Among participants in the pooled mHealth group, 11% met the recommendation to eat more than 7 servings/week of low-fat dairy at the end of the trial. Between-groups difference in mean food groups consumption did not vary markedly between intervention groups, reaching statistical significance only for vegetables and low-fat dairy, although the amount was not clinically relevant (Supplementary Materials, Table S2).

**Figure 2:**
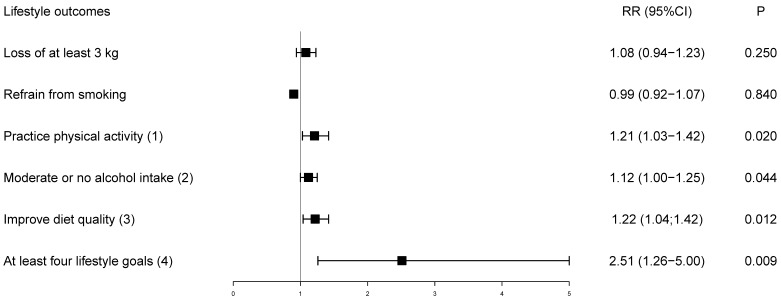
Relative risk of lifestyle goals at the end of the trial adjusted for baseline values. The control group is the reference. (1) Physical activity ≥ 150 min/week; (2) Moderate or no alcohol intake: 100 g (women)/200 g (men)/week; (3) Following two of six dietary recommendations (fruits and vegetables ≥ 21 servings/week; whole grains ≥ 14 servings/week; low-fat dairy ≥ 7 servings/week; sodium-rich foods ≤ 1 serving/week; fast or fried food < 1 serving/week; fried or fatty meats < 1 serving/week); (4) Achieving four out of five lifestyle goals in the follow-up; RR = relative risk.

Figure 2 presents the relative risk for achieving the goals adjusted for baseline values. Although the loss of at least 3 kg and refrain from smoking were not individually effective, achievement of four of five lifestyle goals more than doubled among participants receiving the pooled mHealth intervention (Figure 2). At six months, 70 (41.9%) participants in the mHealth intervention achieved four of five lifestyle goals, while only seven (16.7%) achieved the goal in the control, resulting in an absolute risk reduction of 24.9% (4.7; 45.1) favoring the intervention group.

The Supplementary Materials (Table S3) indicates that none of the individual interventions (TELEM, TELEMEV, and TELEM-TELEMEV) had a statistically significant impact on achieving a weight loss of at least 3 kg or on quitting smoking. However, the other recommendations, such as increasing physical activity, moderating alcohol consumption, and improving diet quality, showed some positive effects. The TELEM and TELEM-TELEMEV interventions were successful in achieving the goal of changing at least four lifestyle habits, but for the TELEMEV intervention, the association was barely borderline. Therefore, the results seemed homogeneous enough to pool the intervention groups. The Supplementary Materials (Table S4) shows that the pooled mHealth had a quite similar effect with statistically significant associations for practice of physical activity, moderate or no alcohol intake, and improved diet quality. Overall, achieving at least four lifestyle goals was 2.5 times more likely for participants in the pooled mHealth group.

### 3.2. Anthropometric Characteristics

Table 3 shows baseline and follow-up anthropometric characteristics and between-groups difference at 6 months. The between-group difference at 6 months reached clinically relevant reduction, but was marginally significant, favoring the pooled mHealth group for body fat (−4.05 kg 95% CI: −8.14; 0.03, *p* = 0.052), segmental trunk fat (−1.69 kg 95% CI: −3.50; 0.12, *p* = 0.067), and WC (−4.36 cm 95% CI: −8.81; 0.082, *p* = 0.054).

## 4. Discussion

This study showed that a lifestyle program with reinforcement using mHealth interventions based on BP monitoring and text messages promoted a statistically significant and clinically relevant greater adherence to achieving at least four lifestyle goals than the UCT alone. Participants who received the mHealth intervention more than double the likelihood of achieving the lifestyle goals. More significant adherence to lifestyle changes in the mHealth group produced a marginally significant but clinically relevant reduction of more than 4 kg in total body fat, 1.7 in segmental trunk fat, and 4 cm in WC.

Several randomized controlled trials in different populations investigated lifestyle changes using mHealth strategies to prevent and control chronic diseases [24,25,26,27]. A lifestyle-focused text messaging program promoted risk factor modification among patients with coronary heart disease [28]. In a cluster-randomized trial where tailored lifestyle text messages were sent for 18 months, Poggio et al. found results somewhat similar to the present study, showing effectiveness for increasing fruit and vegetable intake and physical activity with no effect on alcohol consumption, smoking, or weight loss [29]. Although the intervention group showed greater adherence to lifestyle changes, there was no significant reduction in weight observed in this study. However, there was a marginally significant, but clinically relevant reduction in body fat, segmental trunk fat, and WC. The substitution of certain dietary components, such as increasing the consumption of vegetables and low-fat dairy products while reducing intake of higher-fat foods, has been found to result in decreased anthropometric measurements. It is important to note that this effect does not necessarily promote weight loss.

A similar study used text messages and lifestyle educational sessions versus educational sessions alone in a weight loss program and did not show between-group differences in weight or body fat percentage [30]. The use of mHealth to promote weight loss has been studied with different types of interventions and control groups [31]. Meaningful results in weight loss were seen for interventions compared to nonactive control groups, such as being on the waitlist [31].

With or without mHealth support, BP self-monitoring has effectively promoted BP control, at the expense of medication titration or increased adherence to BP-lowering medications [15,32,33]. However, its effect has never been explored regarding adherence to lifestyle change. This study aimed to improve the lifestyle by adding different approaches. McManus et al. evaluated weight loss in a self-monitoring BP with a digital intervention versus usual care [34]. They also did not observe a difference in the weight measurement (mean difference −0.36 kg, 95% CI: −1.10 to 0.38 kg).

This study shows that, in addition to face-to-face lifestyle intervention, an application based on BP monitoring with a simple text message system is feasible and yields higher lifestyle adherence in patients with uncontrolled hypertension. Current care demands for lifestyle promotion and disease prevention require a long time from health professionals, compromising feasibility in clinical practice. In this scenario, mHealth programs can be an essential ally [35].

To our knowledge, this is the first trial evaluating a lifestyle intervention supported by mHealth to promote BP control and change of lifestyle (without drug titration) in a population of participants with uncontrolled hypertension. We hypothesized that incorporating mHealth into outpatient clinics would lead to shorter consultation times, longer intervals between consultations, and greater adherence to a healthy lifestyle if it proves to be effective. Patients came from primary care clinics, and the findings show the potential of large-scale technology use in public health programs in developing countries.

This study has limitations that should be taken into account when interpreting the results. Our hypothesis was that both mHealth approaches would be more effective than UCT, and the high retention rate of over 90% provided sufficient statistical power to test this. The study was designed to provide insights applicable to patients in public health facilities, and the use of mHealth resources could be a promising alternative to improve adherence to lifestyle changes. However, we lacked the statistical power to determine which mHealth intervention yielded better results, and the availability of such resources may vary. Self-reported information was obtained using validated questionnaires applied by certified researchers, which should minimize potential information biases. The six-month follow-up is relatively long among controlled trials evaluating the effectiveness of mHealth technology. This follow-up period captures the highest level of adherence and effect of the intervention; nevertheless, longer-term studies are necessary to assess sustained interventions.

## 5. Conclusions

A six-month lifestyle intervention supported by application-based BP monitoring and text messages significantly improves adherence to lifestyle goals and is likely to reduce some anthropometric characteristics in comparison with the control without technology support.

## Figures and Tables

**Table 1:** Baseline characteristics, mean ± SD or n (%).

Characteristics	All Participants	Pooled mHealth Group	UCT Group
	(*n* = 231)	(*n* = 186)	(*n* = 45)
Age (years)	55.4 ± 9.5	55.5 ± 9.7	55.0 ± 8.7
Male	120 (51.9)	94 (50.5)	26 (57.8)
White skin color	150 (64.9)	120 (64.5)	30 (66.7)
Education (years)	11.1 ± 3.9	11.1 ± 4.0	11.1 ± 3.9
Currently working	154 (66.7)	124 (66.7)	30 (66.7)
Smoking status			
Never	111 (48.1)	90 (48.4)	21 (46.7)
Former	86 (37.2)	70 (37.6)	16 (35.6)
Current	34 (14.7)	26 (14.0)	8 (17.8)
BMI (kg/m^2^)	30.0 ± 5.1	29.5 ± 4.9	31.3 ± 6.0
Diabetes mellitus	47 (20.3)	37 (19.9)	10 (22.2)
Systolic blood pressure (mmHg)	143.7 ± 11.4	143.5 ± 11.4	144.5 (11.3)
Diastolic blood pressure (mmHg)	89.6 ± 8.1	89.2 ± 8.0	91.3 ± 8.1

Data from independent samples *t*-test for continuous and chi-square for categorical variables; mHealth = mobile health; UCT = usual care treatment; SD = standard deviation.

**Table 2:** Relative risk (95% CI) for achieving the lifestyle goals at the end of the trial by intervention groups, adjusted for baseline values.

	Pooled mHealth Group *n* = 174		UCT Group *n* = 42		RR (95% CI) *
	Baseline	6 Months	Baseline	6 Months	
Weight (kg)	82.7 ± 17.2	81.7 ± 17.2	88.4 ± 21.37	87.6 ± 21.5	−0.39 (−1.49; 0.70) ^a^
No smoking	148 (86.5)	149 (87.1)	34 (81.0)	35 (83.3)	0.99 (0.92; 1.07)
Physical activity ≥ 150 min/week	112 (64.0)	116 (67.1)	21 (50.0)	18 (42.9)	1.21 (1.03; 1.42)
Moderate or no alcohol intake ≤100 g (women)/≤200 g (men)/week	143 (82.7)	158 (91.3)	38 (90.5)	35 (83.3)	1.12 (1.00; 1.25)
Following ≥ two of six dietary recommendations	45 (26.3)	97 (56.7)	13 (31.0)	16 (38.1)	1.22 (1.04; 1.42)
Fruits and vegetables ≥21 servings/week	55 (32.0)	95 (55.2)	13 (31.0)	18 (42.9)	1.12 (0.97; 1.30)
Whole grains ≥14 servings/week	23 (13.4)	47 (27.3)	4 (9.5)	7 (16.7)	1.10 (0.97; 1.24)
Low-fat dairy ≥7 servings/week	14 (8.0)	39 (22.3)	4 (9.5)	5 (11.9)	1.11 (1.00; 1.24)
Sodium-rich foods ≤1 serving/week	19 (11.1)	38 (22.2)	2 (4.8)	7 (16.7)	1.05 (0.92; 1.20)
Fast or fried food <1 serving/week	34 (19.9)	61 (35.7)	10 (23.8)	14 (33.3)	1.03 (0.88; 1.20)
Fried or fatty meats <1 serving/week	19 (11.0)	51 (29.7)	7 (16.7)	11 (26.2)	1.05 (0.90; 1.22)

* Data from the Generalized Poisson mixed adjusted for baseline values; ^a^ Generalized linear models adjusted for baseline value were used for between-groups difference (95% CI) at 6 months; mHealth = mobile health; UCT = usual care treatment.

**Table 3:** Baseline and follow-up anthropometric characteristics and between-groups difference at 6 months [mean (95% CI)].

	Pooled mHealth Group *n* = 186		UCT Group *n* = 45		Between-Groups Difference (95% CI) at 6 Months *	*p* for Between-Groups Difference at 6 Months **
Body Fat Outcomes	Baseline	6 Months	Baseline	6 Months		
Body fat mass (kg)	28.8 (27.5; 30.2)	28.1 (26.7; 29.5)	31.8 (28.3; 35.4)	32.3 (28.3; 36.3)	−4.05 (−8.14; 0.03)	0.052
Percentage of body fat (%)	34.8 (33.6; 36.1)	34.3 (33.0; 35.5)	36.0 (33.8; 38.2)	36.4 (34.0; 38.7)	−2.11 (−4.80; 0.56)	0.122
Segmental trunk fat (kg)	15.2 (14.5; 15.9)	14.8 (14.1; 15.5)	16.6 (14.9; 18.2)	16.5 (14.9; 18.2)	−1.69 (−3.50; 0.12)	0.067
Segmental trunk fat (%)	36.1 (35.0; 37.2)	35.6 (34.5; 36.7)	37.3 (35.4; 39.1)	37.9 (35.5; 39.5)	−1.91 (−4.18; 0.35)	0.098
BMI (kg/m^2^)	29.9 (29.2; 30.6)	29.5 (28.8; 30.2)	31.3 (29.6; 33.1)	31.1 (29.2; 32.9)	−1.56 (−3.49; 0.37)	0.113
Waist circumference (cm)	102.7 (100.9; 104.5)	101.1 (99.4; 102.9)	105.6 (101.7; 109.4)	105.5 (101.7; 110.3)	−4.36 (−8.81; 0.082)	0.054

Data are expressed as mean (95% CI); mHealth = mobile health; UCT = usual care treatment; * Between-groups difference estimated by generalized estimating equation. ** *p*-value for between-groups difference at 6 months, adjusted for Bonferroni.

## Data Availability

The data that support the findings of this study are available from the corresponding author, C.N.D., upon reasonable request.

## Supplementary Materials

The following supporting information can be downloaded at: https://www.mdpi.com/article/10.3390/healthcare11081069/s1, Table S1: Examples of text messages; Table S2: Consumption of each food group per week at baseline and follow-up (mean ± SD) and between-groups difference (95% CI) at 6 months; Table S3: Relative risk for achieving the lifestyle goals at the end of the trial by individual randomization group, adjusted for baseline values; Table S4: Baseline and 6-month lifestyle goals and relative risk (95% CI) for reaching that goal at the end of the trial, adjusted for baseline values.
