# Supplementary material for: Effect of Mobile Health Interventions on Lifestyle and Anthropometric Characteristics of Uncontrolled Hypertensive Participants: Secondary Analyses of a Randomized Controlled Trial
Source: Healthcare (Basel). 2023 Apr 8;11(8):1069. doi: 10.3390/healthcare11081069 (PMC10138120; doi:10.3390/healthcare11081069)
Supplement: Supplementary file 1 [file healthcare-11-01069-s001.zip › healthcare-2260993-supplementary.pdf]

Supplemental Table S1. Examples of text messages.

Type (frequency)	Examples
DASH-type diet (1/week)	Hi <NAME>, healthy eating means at least five servings of vegetables and two servings of fruit every day.
	Hello <NAME>, try to choose poultry or meat trimmed of visible fat and without the skin.
	<NAME>, healthy eating includes ingesting 20 to 30 grams of fiber daily. For this, prefer rice, pasta, and whole-grain bread.
Reduced sodium consumption (1/week)	Hello <NAME>, try avoiding adding salt to your foods by using spices or herbs.
	Hello <NAME>, reducing the consumption of processed foods contributes to lowering blood pressure.
	Hi <NAME>, so as not to overdo the salt consumption, avoid leaving the salt shaker on the table. The food already contains the necessary salt!
Physical activity (1/week)	Hello <NAME>, start practicing physical activity at low intensity and gradually increase.
	Hi <NAME>, make your physical activity enjoyable, invite a friend, neighbor, or spouse to exercise with you.
	Hi <NAME>, did you know that regular physical activity helps to reduce blood pressure, control weight, and reduce stress?
General motivational messages for lifestyle and medications adherence (1/week)	Hello <NAME>, if you are trying to have healthy habits and have a bad day, don't give up. Keep trying.
	<NAME>, did you know that staying at the right weight and eating healthy reduces your risk of having a cardiovascular problem?
	Hi <NAME>, your good health is important. Please try to do more exercise. Activities that make you sweat or your heart beat faster are good for you.

Supplemental Table S2. Consumption of each food group per week at baseline and follow-up (mean ± SD) and between groups difference (95% CI) at 6 months.

Food group	Pooled mHealth group		UCT group		Between groups adjusted difference* (95% CI) at 6 months
	Baseline	6 Months	Baseline	6 Months	
Low-fat dairy	1.29 ± 3.02	3.15 ± 4.30	1.33 ± 3.16	1.92 ± 4.53	1.22 (0.003;2.43)
Whole grains	4.47 ± 7.13	7.92 ± 7.11	3.80 ± 5.41	6.81 ± 6.03	0.887 (-1.00;2.77)
Fruits	7.70 ± 6.91	9.41 ± 5.82	7.31 ± 6.32	9.76 ± 7.21	-0.49 (-2.56;1.57)
Vegetables	10.60 ± 6.99	13.10 ± 7.32	10.82 ± 7.08	10.54 ± 5.32	2.86 (1.17;4.55)
Fast foods or fried foods	1.93 ± 1.59	1.49 ± 1.89	2.04 ± 1.93	1.38 ± 1.46	0.15 (-0.40;0.70)
Fried or fatty meats	4.13 ± 3.60	2.40 ± 2.78	3.44 ± 3.21	2.81 ± 3.25	-0.56 (-1.55;0.43)
Sodium-rich foods	8.25 ± 6.17	5.02 ± 4.78	9.07 ± 6.29	5.43 ± 4.37	-0.15 (-1.51;1.22)

* Generalized linear models, adjusted for baseline values.

Supplemental Table S3. Relative risk for achieving the lifestyle goals at the end of the trial by individual randomization group, adjusted for baseline values.

**Lifestyle goals**	***n***	**Baseline prevalence *n* (%)**	**6 Months prevalence *n* (%)**	**RR (95% CI)***	**P-value**
Loss of at least 3 kg					
TELEMEV$^{\beta}$	43	-	9 (20.9)	1.32 (0.53;3.27)	0.548
TELEM┼	43	-	11 (25.6)	1.66 (0.70;3.95)	0.253
TELEM-TELEMEV$^{£}$	88	-	20 (22.7)	1.50 (0.67;3.35)	0.32
UCT	42	-	7 (16.7)	Ref.	-
Refrain from smoking					
TELEMEV$^{\beta}$	43	36 (76.6)	35 (81.4)	0.99 (0.87;1.13)	0.943
TELEM┼	43	41 (93.2)	41 (95.3)	1.02 (0.93;1.24)	0.662
TELEM-TELEMEV$^{£}$	85	83 (87.4)	73 (85.9)	0.97 (0.89;1.06)	0.534
UCT	42	37 (82.2)	35 (83.3)	Ref.	-
Practice physical activity (1)					
TELEMEV$^{\beta}$	44	29 (61.7)	29 (65.9)	1.40 (0.96;2.05)	0.083
TELEM┼	43	32 (72.7)	32 (74.4)	1.51 (1.03;2.21)	0.035
TELEM-TELEMEV$^{£}$	86	55 (57.9)	55 (64.0)	1.43 (0.99;2.06)	0.055
UCT	42	23 (51.5)	18 (42.9)	Ref.	-
Moderate or no alcohol intake (2)					
TELEMEV$^{\beta}$	44	39 (86.7)	39 (88.6)	1.08 (0.93;1.26)	0.291
TELEM┼	43	34 (79.1)	39 (90.7)	1.15 (0.99;1.33)	0.061
TELEM-TELEMEV$^{£}$	86	74 (83.1)	80 (93.0)	**1.16 (1.01;1.33)**	**0.033**
UCT	42	38 (90.5)	35 (83.3)	Ref.	-
Improve diet quality (3)					
TELEMEV$^{\beta}$	44	15 (34.1)	25 (56.8)	1.46 (0.95;2.26)	0.083
TELEM┼	42	8 (19.0)	23 (54.8)	1.54 (0.93;2.46)	0.071
TELEM-TELEMEV$^{£}$	85	22 (25.9)	49 (57.6)	**1.56 (1.03;2.34)**	**0.033**
UCT	42	13 (31.0)	16 (38.1)	Ref.	-
At least four lifestyle goals (4)					
TELEMEV$^{\beta}$	42	7 (15.9)[a]	13 (30.9)	1.86 (0.84;4.08)	0.124
TELEM┼	42	5 (11.9)[a]	22 (52.4)	**3.20 (1.54;6.65)**	**0.002**
TELEM-TELEMEV$^{£}$	83	13 (15.3)[a]	35 (42.1)	**2.50 (1.23;5.10)**	**0.011**
UCT	42	6 (14.3)[a]	7 (16.7)	Ref.	-

*Data from the Generalized Poisson mixed model adjusted for baseline values.

The control group (without technology) was the reference group. (1) Physical activity ≥150 min/week; (2) Moderate or no ethanol intake: 100g (women)/200g (men) /week; (3) Following two of six dietary recommendations (Fruits and vegetables ≥21 servings/week; Whole grains ≥14 servings/week; Low-fat dairy ≥7 servings/week; Sodium-rich foods ≤1 serving/week; Fast or fried food <1 serving/week; Fried or fatty meats <1 serving/week); (4) Achieving four out of five lifestyle goals in the follow-up; [a] Follow 4 lifestyle (Refrain from smoking, Moderate or no alcohol intake, Improve diet quality) at baseline; RR = relative risk; β personalized text messages to stimulate lifestyle changes via a mobile application; ┼ an automatic oscillometric device to measure and register blood pressure via a mobile application; £ both mHealth interventions; UCT= usual care treatment (without technology).

Supplemental Table S4. Baseline and 6 months lifestyle goals and relative risk (95% CI) for reaching that goal at the end of the trial, adjusted for baseline values.

**Lifestyle goals**	***n***	**Baseline prevalence**	**6 Months prevalence**	**RR (95% CI)***	**P-value**
Loss of at least 3 kg					
Pooled mHealth	174	-	40 (23.0)	1.08 (0.94;1.23)	0.250
UCT	42	-	7 (16.7)	Ref.	-
Refrain from smoking					
Pooled mHealth	171	148 (86.5)	149 (87.1)	0.99 (0.92;1.07)	0.840
UCT	42	34 (81.0)	35 (83.3)	Ref.	-
Practice physical activity (1)					
Pooled mHealth	173	112 (64.0)	116 (67.1)	**1.21 (1.03;1.42)**	**0.020**
UCT	42	21 (50.0)	18 (42.9)	Ref.	-
Moderate or no alcohol intake (2)					
Pooled mHealth	173	143 (82.7)	158 (91.3)	**1.12 (1.00;1.25)**	**0.044**
UCT	42	38 (90.5)	35 (83.3)	Ref.	-
Improve diet quality (3)					
Pooled mHealth	171	45 (26.3)	97 (56.7)	**1.22 (1.04;1.42)**	**0.012**
UCT	42	13 (31.0)	16 (38.1)	Ref.	-
At least four lifestyle goals (4)					
Pooled mHealth	167	25 (14.6)[a]	70 (41.9)	**2.51 (1.26;5.00)**	**0.009**
UCT	42	6 (14.3)[a]	7 (16.7)	Ref.	-

*Data from the Generalized Poisson mixed model adjusted for baseline values. The control group (without technology) was the reference group. (1) Physical activity ≥150 min/week; (2) Moderate or no ethanol intake: 100g (women)/200g (men) /week; (3) Following two of six dietary recommendations (Fruits and vegetables ≥21 servings/week; Whole grains ≥14 servings/week; Low-fat dairy ≥7 servings/week; Sodium-rich foods ≤1 serving/week; Fast or fried food <1 serving/week; Fried or fatty meats <1 serving/week); (4) Achieving four out of five lifestyle goals in the follow-up; [a] Follow 4 lifestyle (Refrain from smoking, Moderate or no alcohol intake, Improve diet quality) at baseline; UCT= usual care treatment (without technology); RR = relative risk.
